# How Frequently Do the Results from Completed US Clinical Trials Enter the Public Domain? - A Statistical Analysis of the ClinicalTrials.gov Database

**DOI:** 10.1371/journal.pone.0101826

**Published:** 2014-07-15

**Authors:** Hiroki Saito, Christopher J. Gill

**Affiliations:** 1 Department of Medicine, Carney Hospital, Dorchester, Massachusetts, United States of America; 2 Center for Global Health and Development, Boston University, Boston, Massachusetts, United States of America; 3 Department of International Health, Boston University School of Public Health, Boston, Massachusetts, United States of America; World Health Organization, Switzerland

## Abstract

**Background:**

Achieving transparency in clinical trials, through either publishing results in a journal or posting results to the ClinicalTrials.gov (CTG) web site, is an essential public health good. However, it remains unknown what proportion of completed studies achieve public disclosure of results (PDOR), or what factors explain these differences.

**Methods:**

We analyzed data from 400 randomly selected studies within the CTG database that had been listed as ‘completed’ and had at least four years in which to disclose results. Using Kaplan-Meier curves, we calculated times from completion to PDOR (defined as publishing the primary outcomes in a journal and/or posting results to CTG), and identified explanatory variables predicting these outcomes using Cox proportional hazards models.

**Findings:**

Among the 400 clinical trials, 118 (29.5%) failed to achieve PDOR within four years of completion. The median day from study completion to PDOR among 282 studies (70.5%) that achieved PDOR was 602 days (mean 647 days, SD 454 days). Studies were less likely to achieve PDOR if at earlier stages (phase 2 vs. phase 3/4, adjusted HR 0.60, 95% CI 0.47–0.78), if they only included adult subjects (adjusted HR 0.61, 95% CI 0.45–0.83), involved randomization (adjusted HR 0.62, 95% CI 0.46–0.83), or had smaller sample sizes (≤50 subjects vs. >50, adjusted HR 0.60, 95% CI 0.44–0.83). Industry-funded studies were significantly less likely to be published than non-industry or blended studies (adjusted HR 0.49, 95% CI 0.36–0.66).

**Conclusions:**

A significant proportion of completed studies did not achieve PDOR within the four years of follow-up, particularly smaller studies at earlier stages of development with industry funding. This constitutes reporting bias and threatens the validity of the clinical research literature in the US.

## Introduction

Transparency is of paramount importance in clinical trials. The public must be informed if the premise of a clinical trial was confirmed or invalidated, and expects that, once a study involving human subjects is completed, its results will be published in the medical literature or posted to some other open-access platform. Failure of transparency conflicts with the bedrock ethical principles governing clinical research (justice, beneficence and respect for subjects) [1], and, by risking reporting bias, undermines the validity of the scientific literature. Promoting transparency in clinical trials is an intrinsic public health good.

In the U.S., legislative efforts to promote transparency have been dominated by two Congressional acts. The first was the ‘Food and Drug Administration Modernization Act’ (FDAMA) of 1997, which established the web-based ClinicalTrials.gov (CTG) registry [2]. By requiring clinical trials to register within 21 days of enrolling the first subject, FDAMA made it harder for trials to go unnoticed. However, it did not require reporting of results from completed studies, which is needed to determine the proportion whose results end up in the public domain. In response, in 2007 the US Congress passed the Food and Drug Administration Amendments Act (FDAAA) which required ‘applicable clinical trials’ to post basic study results to CTG within one year of completion [3]. Unfortunately, the definition of ‘applicable clinical trials’ exempted phase 1 studies and studies at any stage of products pre-licensure [4], [5].

From the perspective of achieving transparency, both publishing results in peer-reviewed journals and posting results to CTG is the ideal; conversely, failing to either publish or post results to CTG indicates a failure of transparency. (For the purpose of this manuscript, “publishing” refers to publication in peer-reviewed journals hereafter.) Available evidence suggests that lack of transparency due to undisclosed results has been a problem [6]–[10]. Previously, we reported that among US-based, industry-funded phase 2 and higher clinical trials, less than 25% posted their results to CTG within a year of study completion [11]. Similarly, among National Institutes of Health (NIH) funded trials, 32% remained unpublished after the median follow-up of 51 months from completion [12].

Thus, public disclosure of results (PDOR) is critical to achieving transparency of clinical trials. Publication in peer-reviewed journals is the gold-standard of disclosing results in the scientific community. Similarly CTG is currently the only mandated registry for US based clinical trials. Therefore, for the purposes of this article, we defined PDOR as publishing a study's primary outcomes in a peer-reviewed journal and/or posting results to CTG.

To better characterize the process by which completed studies do (or do not) achieve PDOR, we analyzed the CTG database itself. We addressed the following questions:

1. Of completed studies, what proportion achieved PDOR?
2. What is the average time from study completion to PDOR, and what factors influence PDOR?
3. How did FDAAA, as a way to promote transparency, influence PDOR?

## Methods

### Data Source

The dataset was downloaded from CTG on January 6, 2013. We selected US-based (defined by Food and Drug Administration (FDA) as having one or more sites in the US), interventional studies that had at least one arm of ‘drug’ intervention defined by FDAAA, at phase 2 or beyond, and listed on CTG as completed between January 1 and December 31, 2008. The year 2008 was selected for two reasons: 1) this provided a grace period of at least four years from study completion in which to publish the trial's primary outcomes (Ross et al. previously showed the median time from study completion to publication among published trials was 23 months [12]); and 2) 2008 was the first year that FDAAA's CTG posting requirements came into effect. From this larger set, 400 studies were randomly selected for analysis using a web-based random number generator (http://stattrek.com/statistics/random-number-generator.aspx).

### Transparency of Studies

A completed study was defined as ‘PDOR achieved’ if either publication and/or result posting had occurred by January 6, 2013; conversely a study was deemed ‘failure of PDOR’ if neither condition was met. If both were achieved, the earlier event was used for our ‘time to PDOR’ analyses, calculated by subtracting the publication or posting date from the study completion date. If neither publication nor result posting occurred, time to censoring was calculated by subtracting January 6, 2013 from the study completion date.

### Determinations of Publications in the Peer Reviewed Literature

For each of the 400 completed studies, we determined whether or not it resulted in a publication in a peer-reviewed journal of the study's primary outcome(s). When a study listed more than one primary outcome in CTG and multiple publications were found, the earliest paper presenting at least one primary outcome was selected.

The following two search strategies were used:

1) If a publication was indexed in CTG, the paper was inspected to confirm whether it reported the study's primary outcome as registered on CTG;

2) If no publications were indexed on CTG, we conducted a Boolean search in PubMed (http://www.ncbi.nlm.nih.gov/pubmed/) using the medical condition(s) and/or an intervention(s) studied and/or the name(s) of principle investigator(s) and/or the “Responsible Party”. The earliest publication date was used for our analysis; if the calendar date was not specified, we imputed the first day of the month using the month and year shown in PubMed or the journals.

To validate the accuracy of method 2, we screened all 126 publications found using search method 1: 96.8% (122/126) of the publications were confirmed by method 2.

### Description of Covariates

Variables from the CTG dataset were recoded as follows: subject ages listed on CTG as ‘Adult’, ‘Senior’ or ‘Adult|Senior’ were re-categorized into ‘adult only’; studies including ‘Child’, ‘Adult|Child’ or ‘Child|Adult|Senior’ were re-categorized as ‘child involved’. Study phases were categorized as ‘phase 2’ if listed as ‘Phase 1|Phase 2’ or just ‘Phase 2’, and as ‘phase 3’ if listed as ‘Phase 2|Phase 3’ or just ‘Phase 3’. The number of study subjects was categorized as ‘less than or equal to 50’ (≤50) vs. ‘more than 50’ (>50). Funding source was categorized into ‘funded purely by industry’, ‘funded purely by non-industry sources’ or ‘blended’ for those studies with funding both from industry and non-industry sources. Study designs were categorized into ‘randomized’ vs. ‘non-randomized’. Study investigators were categorized into ‘academia’ vs. ‘non-academia’. The ‘Primary Completion Date’, defined as the date of data collection of primary outcomes from the final study subject, listed in the CTG dataset was used as the study completion date [13]. Since CTG did not include the day of the month for study initiation or completion, we imputed the first day of the month for those dates. Of note, we did not include possibly relevant but unreported covariates such as medical specialties of studies in our analyses: such definitions are post hoc since they would have to be appended by us (not uploaded to CTG by researchers of the studies as with all other covariates) and more subjective decisions would have been involved.

### Sample Size Assumptions

The sample size was calculated from an assumption of difference in proportional hazards of achieving PDOR between industry and non-industry/blended studies as our main outcome: based on a subset of the dataset, we estimated that 60% of industry and 75% of non-industry/blended studies would achieve PDOR over four years, respectively. With an alpha error level of 0.05, 80% power, accrual time of one year and an exponential hazard function, 126 for each group was required. Given the exploratory nature of this analysis, and anticipating that we might need additional power for our multivariate models, we presumptively increased this by ∼50%, rounding up to 400 studies in total.

### Statistical Analysis

For continuous variables, means, medians and standard deviations were obtained. For categorical variables, proportions were obtained. The Kaplan-Meier survival method with the log rank test was used in univariate analyses of survival analyses. Our primary analysis used Cox proportional hazards to model time to PDOR, publication and posting, adjusting for covariates. All analyses were performed using SAS, version 9.3.

## Results

### Sample Characteristics

**Figure 1:**
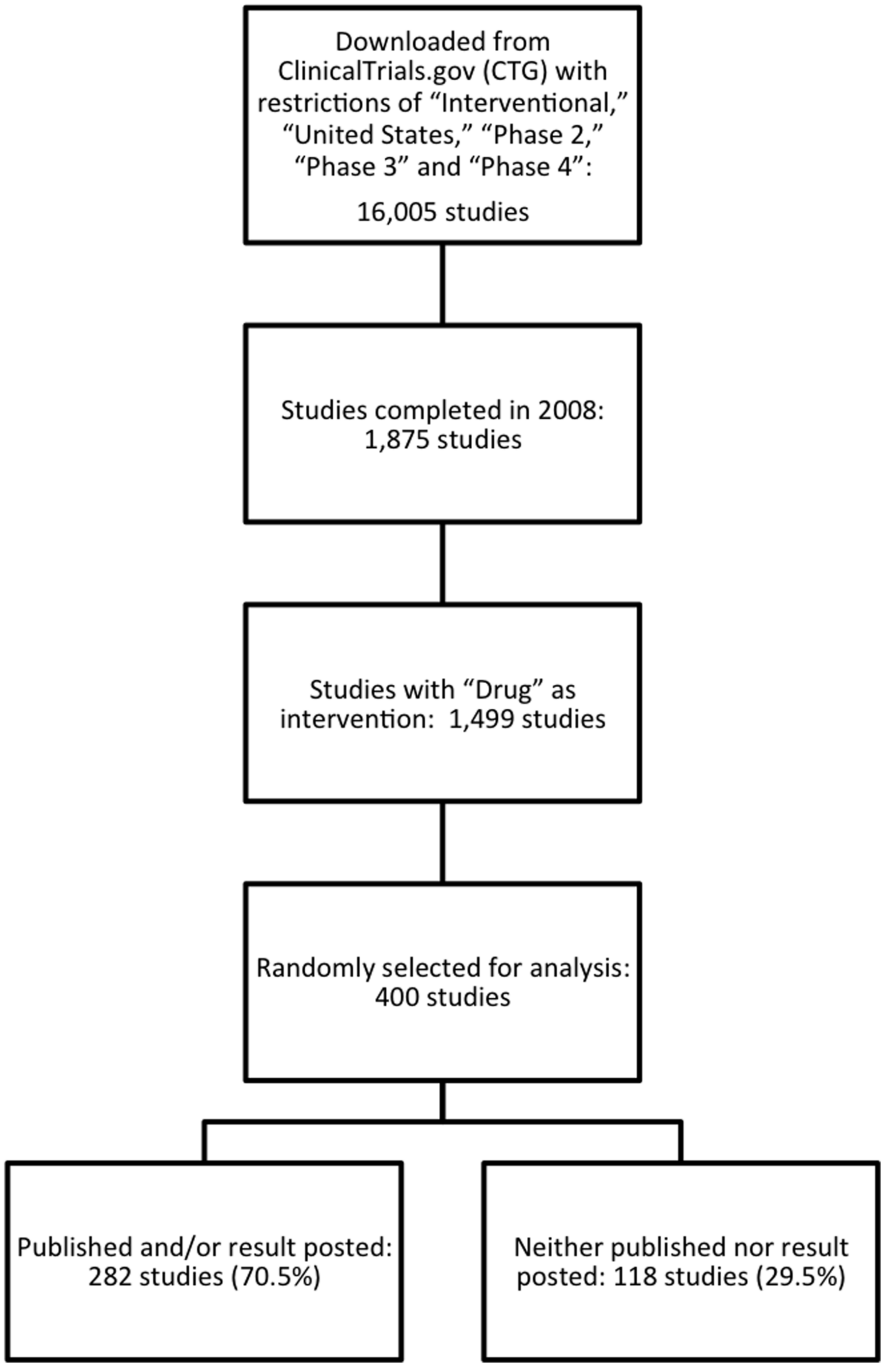
Flow diagram of the data selection. This explains how the clinical trials for the analyses were selected.

1,499 studies registered in CTG with a completion date entered in 2008 met our inclusion criteria, from which 400 were randomly chosen for our analysis (Figure 1) (The dataset is available on-line). The baseline characteristics of these studies are summarized in Table 1. 247 studies (61.7%) were funded purely by industry; 106 studies (26.5%) had no industry funding; and 47 studies (11.8%) had blended funding. A majority (209 studies, 52.3%) were phase 2 studies; 130 studies (32.5%) and 61 studies (15.2%) were phase 3 and 4 studies, respectively.

**Table 1:** Characteristics of the Studies Selected (N = 400).

Characteristics	n (%)
**Funding (n = 400)** *	
Industry	247 (61.7)
Non-industry	106 (26.5)
Blended	47 (11.8)
**Study Phase (n = 400)** *	
Phase 2	209 (52.3)
Phase 3	130 (32.5)
Phase 4	61 (15.2)
**Gender (n = 400)** *	
Both	356 (89.0)
Female only	31 (7.7)
Male only	13 (3.3)
**Age Group (n = 400)**	
Adult only	332 (83.0)
Child involved	68 (17.0)
**Randomization (n = 399)** **	
Randomized	271 (67.9)
Not randomized	128 (32.1)
**Investigator (n = 400)**	
Non-academia	238 (59.5)
Academia	162 (40.5)
**Number of Study Subjects (n = 389)** **	
Median	98
Mean (SD)	249 (452)
Range	1–5993
**Study length (days) (n = 400)**	
Median	639
Mean (SD)	874 (817)
Range	29–6908

*Phase 3 and 4 were aggregated as “phase 3/4”, non-industry funded and blended studies were aggregated as “non-industry or blended” and female only and male only were aggregated as “male or female only” in the analysis.

**Some data were missing in CTG registry and the total number did not sum up 400.

### Proportion of Studies achieving PDOR

Ultimately, 282/400 studies (70.5%) achieved PDOR through publication and/or result posting to CTG (Table 2). 98 studies (24.5%) both published and posted to CTG; 131 studies (32.8%) published but did not post; 53 studies (13.2%) only posted, and 118 (29.5%) neither published nor posted results to CTG. The median time from trial completion to PDOR was 602 days (mean: 647 days, SD 454 days).

**Table 2:** Numbers of completed studies achieving Public Disclosure of Results (PDOR), publication and result posting to ClinicalTrials.gov (CTG) website, and time to do so (N = 400).

	Numbers (%)		Median Days after study completion* (mean, SD)
**PDOR achieved (published and/or posted to CTG)**			
PDOR achieved	282 (70.5)		602 (647, 454)
Failure of PDOR	118 (29.5)		
**Publication**			
Published	229 (57.3)		741 (723, 486)
Not Published	171 (42.7)		
**Posting on CTG**			
Result Posted	151 (37.8)		602 (730, 393)
Not Posted	249 (62.2)		
**Both publication and posting on CTG**		**Median Days of the later event after study completion (mean, SD)** *	
	98 (24.5)		935 (952, 354)

*Results limited to those studies that ever achieved PDOR (publication and/or posting to CTG).

### Factors Predicting PDOR

When the data was analyzed preliminarily, non-industry and blended studies showed very similar results, therefore these two types of studies were combined in the final analyses. Phase 3 and phase 4 studies were also combined for the same reason.

Overall, there was no statistical difference in rate of achieving PDOR between industry and non-industry/blended studies (median days to PDOR: 968 days for industry-funded studies vs. 843 days for non-industry/blended studies, p = 0.21).

After adjusting for funding source, studies were less likely to achieve PDOR if at phase 2 (adjusted HR 0.60, 95% CI 0.47–0.78), were conducted in adults only (adjusted HR 0.61, 95% CI 0.45–0.83), involved randomization (adjusted HR 0.62, 95% CI 0.46–0.83), or had ≤50 subjects (adjusted HR 0.60, 95% CI 0.44–0.83) (Table 3).

**Table 3:** Factors predicting Public Disclosure of Results (PDOR) of completed studies (publication and/or posting to ClinicalTrials.gov (CTG) website).

	Median Days to Transparency (95% CI)	Log-Rank test p value	Adjusted Hazard Ratio (95% CI)
**Funding (n)**			
Non-Industry or Blended (143)	843 (694–1039)	0.21	1
Industry only (241)	968 (785–1126)		0.84 (0.63–1.12)
**Study Phase (n)**			
Phase 3 or 4 (186)	706 (602–789)	<0.001	1
Phase 2 (198)	1176 (1002–1308)		0.60 (0.47–0.78)
**Gender (n)**			
Male or female only (39)	1121 (844 -)	0.16	
Both male and female (345)	883 (744–1002)		
**Age Group (n)**			
Child involved (64)	667.5 (526–833)	<0.001	1
Adult only (320)	992 (870–1174)		0.61 (0.45–0.83)
**Randomization (n)**			
Not randomized (119)	787 (684–947)	0.069	1
Randomized (264)	982 (844–1134)		0.62 (0.46–0.83)
**Investigator (n)**			
Academia (151)	947 (730–1186)	0.89	
Non-academia (233)	888 (751–1072)		
**Number of Study Subjects (n)**			
>50 (254)	827.5 (731–969)	0.008	1
≤50 (121)	1178 (919–1504)		0.60 (0.44–0.83)

**Figure 2:**
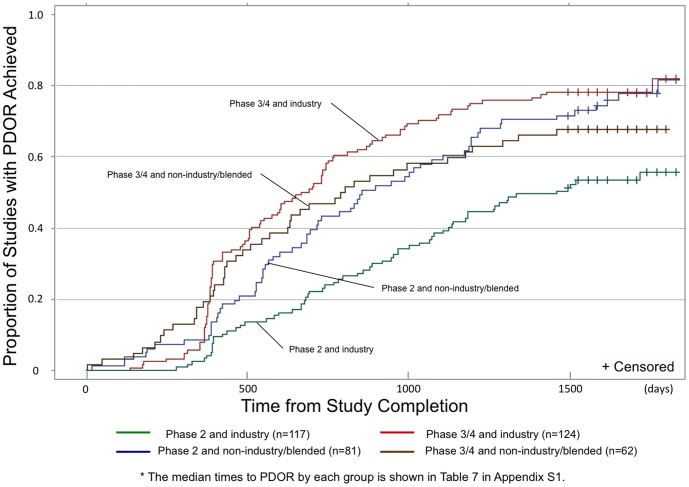
Cumulative rates of Public Disclosure of Results (PDOR) among selected studies categorized by funding source and phase of development (days from study completion to publication and/or posting to ClinicalTrials.gov website). This X axis indicates days from study completion and Y axis indicates proportion of studies that achieved PDOR. - Green line shows phase 2 and industry studies. - Blue line shows phase 2 and non-industry/blended studies. - Red line shows phase 3/4 and industry studies. - Brown line shows phase 3/4 and non-industry/blended studies. * The median times to PDOR by each group is shown in Table S4 in Appendix S1.

Further analysis revealed that the time for industry-funded phase 2 studies to achieve PDOR was significantly longer than those of all other combinations of funding source and study phase (median days to PDOR: 1462 days for industry-funded phase 2 studies vs. 736 days for the other studies, p<0.001) (Figure 2). We noted an inflection point for industry-funded trials, particularly for phase 3/4 studies, occurring about one year after trial completion. Among industry-funded studies, the rate of phase 3/4 studies achieving PDOR was significantly higher than for phase 2 studies (median days to PDOR: 1462 days for phase 2 vs. 679 days for phase 3/4 studies, p<0.001). Conversely, for studies that were not funded solely by industry, time to PDOR had no association with study phase (median days to PDOR: 857 days for phase 2 vs. 797 days for phase 3/4 studies, p = 0.74).

### Publication of Completed Trials

**Figure 3:**
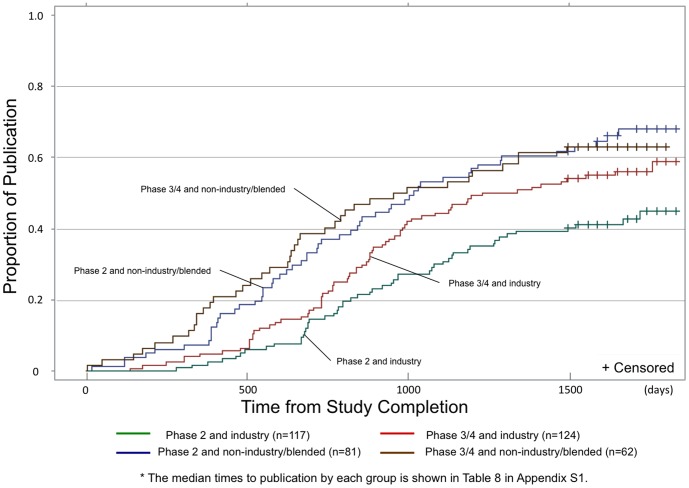
Effect of funding and study phase on proportion of studies that published their primary outcomes in a peer-review journal listed in PubMed by time since study completion. This X axis indicates days from study completion and Y axis indicates proportion of studies that had publication in a peer-review journal. - Green line shows phase 2 and industry studies. - Blue line shows phase 2 and non-industry/blended studies. - Red line shows phase 3/4 and industry studies. - Brown line shows phase 3/4 and non-industry/blended studies. * The median times to publication by each group is shown in Table S5 in Appendix S1.

Studies were less likely to be published and took far longer to be published if funded solely by industry (adjusted HR 0.49, 95% CI 0.36–0.66), or had ≤50 subjects (adjusted HR 0.60, 95% CI 0.43–0.83) (Table S1 in Appendix S1). In addition, publication rates for industry-funded phase 2 studies were significantly lower than for industry-funded phase 3/4 studies (median days to publication: un-measurable for phase 2 studies (because fewer than half were ever published) vs. 1284 days for phase 3/4 studies, p = 0.03) (Figure 3). Conversely, among non-industry funded/blended studies, publication rates did not differ by study phase with 66.3% being published within four years (median days to publication: 1016 days for phase 2 vs. 975 days for phase 3/4 studies, p = 0.99).

### Posting of Completed Trial Data to CTG

**Figure 4:**
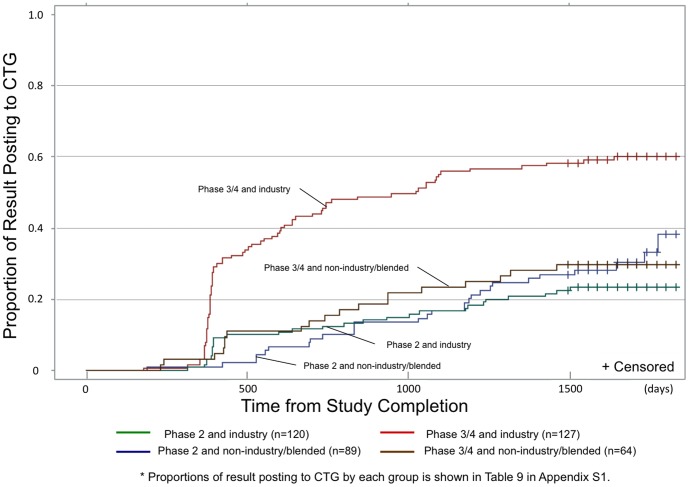
Effect of funding and study phase on proportion of studies that made available their primary outcomes in ClinicalTrials.gov website by time since study completion. This X axis indicates days from study completion and Y axis indicates proportion of studies that posted results to CTG. - Green line shows phase 2 and industry studies. - Blue line shows phase 2 and non-industry/blended studies. - Red line shows phase 3/4 and industry studies. - Brown line shows phase 3/4 and non-industry/blended studies. * Proportions of result posting to CTG by each group is shown in Table S6 in Appendix S1.

A large inflection point in CTG postings among industry-funded studies was again observed about one year from trial completion (Figure 4), and was seen to drive the inflection point seen in the overall time to PDOR analysis (Figure 2). This was particularly noticeable among phase 3/4 studies: ultimately, 58.3% of industry-funded phase 3/4 studies posted results to CTG. As shown in Figure 4, the survival curves for industry and non-industry funded/blended studies crossed around day 400. In order to hold proportionality as an assumption of Cox proportional hazards model, we focused on posting events beyond the first 400 days in our Cox proportional hazards model resulting in exclusion of 52 studies. Stratifying by phase 2 vs. 3/4 phases revealed diametrically opposite patterns in terms of the relationship between posting rates and funding source. Compared with non-industry funded/blended studies, industry-funded phase 2 studies were significantly less likely to post results (adjusted HR 0.47, 95% CI 0.23–0.96), while industry-funded phase 3/4 studies were significantly more likely to post results (adjusted HR 2.25, 95% CI 1.24–4.09) (Tables S2 and S3 in Appendix S1). Of note, 48 out of the excluded 52 studies (92.3%) that posted results to CTG within the initial 400 days were funded solely by industry.

## Discussion

Our analysis suggests that a high proportion of completed US-based human subjects clinical trials did not achieve timely transparency either through publication in peer-review journals or by posting basic results to CTG, or PDOR as defined in this article. Moreover, the probability that a given study achieves PDOR varied significantly as a function of study funding source, phase of development, and other factors: studies were less likely to achieve PDOR if at phase 2, were randomized, involved only adult subjects, and had smaller sample sizes. In this dataset, roughly 30% of clinical studies failed to enter the public domain during the four years of follow-up, with even lower rates for early phase, industry-funded trials among which only about half achieved PDOR. Extrapolating from these rates, of the ∼16,000 interventional, human subjects studies that met our search criteria, we estimate that ∼5,000 failed to achieve PDOR, equating to roughly 500 studies per year. This is conservative, because it excludes phase 1 trials from the denominator.

Considering the two ways of achieving PDOR separately revealed further nuanced differences. For example, among industry-funded studies, CTG posting rates were high for phase 3/4 studies, but extremely low for phase 2 studies. By contrast, non-industry-funded or blended studies displayed similar rates of publication or posting to CTG regardless of study phase, even though results were rarely posted to CTG.

How are these results to be interpreted?

As an overall synthesis, these findings provide strong evidence of reporting bias. Reporting bias is classically defined as a group of biases that induce differential probabilities of publication [14]. Our analyses showed over- and under-representation of studies achieving PDOR, which is considered to be a type of reporting bias. For example, we observed that randomized trials were significantly less likely to achieve PDOR than non-randomized trials. We theorize that this may reflect the fact that randomized trials are hypothesis driven (e.g., “intervention A will be better than intervention B”) and hence less likely to achieve PDOR if the hypothesis is not upheld by the study results. While Lundh et al. have identified industry funding as a risk factor for publishing more favorable results [15], a type of reporting bias, it should be noted that the rate of achieving PDOR by non-industry/blended studies did not differ from that of industry funded studies.

The associations between publication and posting rates and funding source merit further consideration and may reflect differing motivations between each set of researchers. Among academics working within the ‘publish or perish’ model, and who represent the majority of non-industry funded/blended studies, study phase may be less critical than the need to publish research findings in journals. By contrast, given the low rate of publication for industry-funded phase 2 studies despite the fact that the industry generally has the greater resources to conduct research, the industry may publish more results for products that have advanced to phase 3 or beyond and are perceived to be commercially viable. In other words, the decision to publish – or more importantly not to publish – could well be driven by commercial considerations as Johnson et al. documented previously [16]. Conversely, we hypothesize that regulatory requirements, or the fear of sanctions from non-compliance with legislation, may drive the differences observed in rates of posting results to CTG. In support of this theory, among industry-funded phase 3/4 studies, the majority posted their results at about one year from completion, which coincides closely with the deadline established by FDAAA.

What could be done to promote transparency through PDOR?

First, FDAAA exempted a large number of studies from the CTG posting requirement, including phase 1 studies or studies of products pre-licensure. This had the inadvertent effect of creating a legislative blind that systematically conceals much of the medical literature. Rescinding that exemption would be a reasonable step towards promoting transparency. Our study suggests, as opposed to publication, PDOR through posting results to CTG could be achieved in a timely manner but that current incentives (or sanctions for non-compliance) do not appear to be very effective.

Second, our previous research showed how exquisitely sensitive compliance rates were to pressure from non-regulatory actions: the decision of the International Committee of Medical Journal Editors (ICMJE) to embargo all studies that failed to register on CTG boosted registration rates 5-fold within a year [11], [17]. It is worth considering whether transparency could also be improved if the ICMJE refused publication of any study of a product that had not consistently posted results to CTG throughout all phases of its development process (for example, a paper of a phase 2 study would not be considered for publication unless the phase 2 AND the preceding phase 1 studies had been registered in AND posted their results to CTG). Even better would be if the policy were extended to all high impact medical journals.

Third, it would be relatively straightforward to redesign the CTG website to automatically send reminder emails asking investigators to post for studies entered as ‘completed’. Whether this could reduce bias in posting behaviors is a testable question.

Our analysis had several limitations. First, given our decision to offer a 4-year grace period from trial completion, we were necessarily limited to the cohort of studies completed in 2008. It is possible that transparency rates for studies completed in later years might behave differently. However, our analysis included posting and publication events that occurred through 2013, so the behavior of the researchers was not limited to the circumstances present solely in 2008.

Second, as suggested in an earlier publication by Prayle et al., it can be difficult to identify studies that met the criteria for ‘applicable clinical trials’ as defined in FDAAA [8]. However, this only pertains to PDOR via posting to CTG: as a matter of good citizenship, this does not exonerate poor publication rates.

Third, our analysis offered no insight into whether the results of studies themselves influenced PDOR. Many studies were focused on publication bias, a type of reporting bias defined narrowly as over-representation of positive studies in the scientific literature [18]–[20]. More importantly, our analysis clearly shows that a high proportion of results from completed studies never enter the public domain.

Fourth, we did not include all the subsidiary outcomes in the search of publication. Instead, primary outcomes and primary completion dates were used in our analyses. Reporting of study completion date is not mandatory under FDAAA, as opposed to ‘primary completion dates’ and it prevented us from analyzing time to PDOR for other than primary outcomes. While the overall rate of studies that achieved PDOR could be higher with secondary outcomes included in our analyses, it is also possible that some of these primary outcomes could have been revised in CTG over time (making it appear that publishing of the primary outcome had occurred when in fact the primary outcome had been elevated post hoc). This could make it appear in our analyses that the rate of achieving PDOR was better than it actually was.

Fifth, we limited our search to publications listed in PubMed. We justify this on the basis that the majority of US studies are published in journals indexed by PubMed and that PubMed is an open access library and, *de facto*, the lay public's primary access route to the medical literature. Also, we focused only on publications to peer-review journals, which involve more critical assessment of study methods and results, as opposed to other information sources such as conference proceedings and websites of private pharmaceutical companies because they are not necessarily a systematic way to disclose results to the public.

Lastly, our analysis was dependent on the fidelity of the data within CTG: the information entered on CTG is not reviewed by the National Library of Medicine either for completeness or accuracy, so we were forced to take the data entered at face value. The purpose of our paper is rather to quantify the number of clinical trials entering the public domain, than to evaluate the quality of results that entered the public domain. While other investigators have challenged the quality of information entered on CTG [21], this too falls into the category of limitations for which there is no current solution since we are blind to information that may have been hidden.

In conclusion, our analysis shows that failure to PDOR is widespread among US-based human subjects studies, and that observed differential rates of PDOR achieved constitute a form of reporting bias. The scientific literature, and to some extent the CTG registry itself, are a public trust and invaluable common resources. These resources are in jeopardy. Moreover, the current status quo violates our ethical obligation to study subjects who would likely be dismayed to learn that the results from those studies might never enter the public domain. Given the evident need to protect fidelity of the scientific literature and the need to maintain the public's faith in clinical research, it is essential that the research community unite around the common goal of maximizing transparency.

## Supporting Information

Appendix S1 **On-line only tables.**
(DOCX)
