# Supplementary material for: How Frequently Do the Results from Completed US Clinical Trials Enter the Public Domain? - A Statistical Analysis of the ClinicalTrials.gov Database
Source: PLoS One. 2014 Jul 15;9(7):e101826. doi: 10.1371/journal.pone.0101826 (PMC4098992; doi:10.1371/journal.pone.0101826)
Supplement: Appendix S1 — On-line only tables. (DOCX) [file pone.0101826.s001.docx]

**Appendix S1: On-line only tables.**

| **Table S1: Factors related to publication of completed studies** | | | | | | | | | |  |
| --- | --- | --- | --- | --- | --- | --- | --- | --- | --- | --- |
|  | | **Median Days to Publication (95% CI)** | **Log-Rank test**  **p value** | **Adjusted Hazard Ratio (95% CI)** | | |  |  |  |  |
| **Funding (n)** | | | | | | | | | | |
|  | Non-Industry or Blended (143) | 1002  (821 - 1285) | <0.001 | | 1 | | | |  |  |
|  | Industry only (241) | 1719  (1270 - ) |  | | 0.49  (0.36 – 0.66) | | | |  |  |
| **Study Phase (n)** | | | | | | | | | | |
|  | Phase 3 or 4 (186) | 1188  (976 - 1493) | 0.17 | |  | | | |  |  |
|  | Phase 2 (198) | 1520  (1188 - ) |  | | |  | | |  |  |
| **Gender (n)** | | | | | | | | | | |
|  | Male or female only (39) | -  (1188 - ) | 0.12 | |  | | |  |  |  |
|  | Both male and female (345) | 1282  (1084 - 1583) |  | |  | | |  |  |  |
| **Age Group (n)** | | | | | | | | | | |
|  | Child involved (64) | 1103  (843 - 1655) | 0.21 | |  | | |  |  |  |
|  | Adult only (320) | 1398.5  (1186 - 1757) |  | | |  | | |  |  |
| **Randomization (n)** | | | | | | | | | | |
|  | Not randomized (119) | 1186  (843 - 1461) | 0.044 | |  | | |  |  |  |
|  | Randomized (264) | 1494.5  (1181 - ) |  | |  | | |  |  |  |
| **Investigator (n)** | | | | | | | | | | |
|  | Academia (151) | 1107  (898 - 1343) | 0.012 | |  | | |  |  |  |
|  | Non-academia (233) | 1617  (1230 - ) |  | |  | | |  |  |  |
| **Number of Study Subjects (n)** | | | | | | | | | | |
|  | > 50 (254) | 1213.5  (1042 - 1551) | 0.23 | | 1 | | |  |  |  |
|  | ≤ 50 (121) | 1655  (1199 - ) |  | | 0.60  (0.43 – 0.83) | | |  |  |  |

| **Table S2: Factors related to results posting to ClinicalTrials.gov (CTG) website among completed phase 2 studies** | | | | | | | | | |  |
| --- | --- | --- | --- | --- | --- | --- | --- | --- | --- | --- |
|  | | **% Non-result posting**  **4 years after study completion**  **(± SE)** | **Log-Rank test**  **p value*** | **Adjusted Hazard Ratio (95% CI)*** | | |  |  |  |  |
| **Funding (n)** | | | | | | | | | | |
|  | Non-Industry or Blended (89) | 73.0 (± 4.7) | 0.013 | | 1 | | | |  |  |
|  | Industry only (120) | 78.3 (± 3.8) |  | | 0.47  (0.23 – 0.96) | | | |  |  |
| **Gender (n)** | | | | | | | | | | |
|  | Male or female only (31) | 77.4 (7.5) | 0.30 | |  | | | |  |  |
|  | Both male and female (178) | 75.8 (3.2) |  | | |  | | |  |  |
| **Age Group (n)** | | | | | | | | | | |
|  | Child involved (29) | 86.2 (± 6.4) | 0.49 | |  | | |  |  |  |
|  | Adult only (180) | 74.4 (± 3.3) |  | |  | | |  |  |  |
| **Randomization (n)** | | | | | | | | | | |
|  | Not randomized (93) | 71.0 (± 4.7) | 0.024 | | 1 | | |  |  |  |
|  | Randomized (115) | 80.0 (± 3.7) |  | | | 0.48  (0.24 – 0.99) | | |  |  |
| **Investigator (n)** | | | | | | | | | | |
|  | Academia (97) | 74.2 (± 4.4) | 0.041 | |  | | |  |  |  |
|  | Non-academia (112) | 77.7 (± 3.6) |  | |  | | |  |  |  |
| **Number of Study Subjects (n)** | | | | | | | | | | |
|  | > 50 (112) | 68.8 (± 4.4) | 0.34 | | 1 | | |  |  |  |
|  | ≤ 50 (91) | 83.5 (± 3.9) |  | | 0.43  (0.22 – 0.81) | | |  |  |  |

* Studies that posted results prior to 400 days excluded from analysis

| **Table S3: Factors related to results posting to ClinicalTrials.gov (CTG) among completed phase 3/4 studies** | | | | | | | | | |  |
| --- | --- | --- | --- | --- | --- | --- | --- | --- | --- | --- |
|  | | **% Non-result posting**  **4 years after study completion**  **(± SE)** | **Log-Rank test**  **p value*** | **Adjusted Hazard Ratio (95% CI)*** | | |  |  |  |  |
| **Funding (n)** | | | | | | | | | | |
|  | Non-Industry or Blended (64) | 71.9 (± 5.6) | 0.035 | | 1 | | | |  |  |
|  | Industry only (127) | 41.7 (± 4.4) |  | | 2.25  (1.24 – 4.09) | | | |  |  |
| **Gender (n)** | | | | | | | | | | |
|  | Male or female only (13) | 61.5 (13.5) | 0.96 | |  | | | |  |  |
|  | Both male and female (178) | 51.1 (3.8) |  | | |  | | |  |  |
| **Age Group (n)** | | | | | | | | | | |
|  | Child involved (39) | 33.3 (± 7.6) | <0.001 | | 1 | | |  |  |  |
|  | Adult only (152) | 56.6 (± 4.0) |  | | 0.31  (0.17 – 0.55) | | |  |  |  |
| **Randomization (n)** | | | | | | | | | | |
|  | Not randomized (35) | 54.3 (± 8.4) | 0.74 | |  | | |  |  |  |
|  | Randomized (156) | 50.6 (± 4.0) |  | | |  | | |  |  |
| **Investigator (n)** | | | | | | | | | | |
|  | Academia (65) | 69.2 (± 5.7) | 0.034 | |  | | |  |  |  |
|  | Non-academia (126) | 42.9 (± 4.4) |  | |  | | |  |  |  |
| **Number of Study Subjects (n)** | | | | | | | | | | |
|  | More than 50 (152) | 46.1 (± 4.0) | 0.080 | |  | | |  |  |  |
|  | Less than or equal to 50 (34) | 70.6 (± 7.8) |  | |  | | |  |  |  |

* Studies that posted results prior to 400 days excluded from analysis

Table S4. Median days to Public Disclosure of Results (PDOR) among selected studies categorized by funding source and phase of development (days from study completion to publication and/or posting to ClinicalTrials.gov website)

| **Study Phase and Funding source (n)** | **Median Days to**  **PDOR (95% CI)** | **Log Rank test**  **p value** |
| --- | --- | --- |
| Phase 2 and Industry (117) | 1,462 (1,135 - ) | < 0.001 |
| Phase 3/4 and Industry (124) | 679 (538 - 751) |  |
| Phase 2 and Non-Industry/Blended (81) | 857 (696 – 1,107) |  |
| Phase 3/4 and Non-Industry/Blended (62) | 797 (546 – 1,199) |  |

Table S5. Median days from study completion to publication in a peer-reviewed journal listed in PubMed among selected studies categorized by funding source and phase of development

| **Study Phase and Funding (n)** | **Median Days to**  **Publication (95% CI)** | **Log Rank test**  **p value** |
| --- | --- | --- |
| Phase 2 and Industry (117) | No median* (1,520 - ) | <0.001 |
| Phase 3/4 and Industry (124) | 1,284 (988 - ) |  |
| Phase 2 and Non-Industry/Blended (81) | 1,016 (821 – 1,291) |  |
| Phase 3/4 and Non-Industry/Blended (62) | 975 (659 – 1,493) |  |

*Fewer than half of this category were published, therefore the median days could not be computed.

Table S6. Proportions of studies whose results of primary outcomes were not available in ClinicalTrials.gov website within 4 years

| **Study Phase and Funding (n)** | **% not posting to CTG within 4 years of study completion**  **(± SE)*** | | **Log Rank test**  **p value** |  |
| --- | --- | --- | --- | --- |
| Phase 2 and Industry (120) | | 78.3 (± 3.8) | < 0.001 | |
| Phase 3/4 and Industry (127) | | 41.7 (± 4.4) |  | |
| Phase 2 and Non-Industry/Blended (89) | | 73.0 (± 4.7) |  | |
| Phase 3/4 and Non-Industry/Blended (64) | | 71.9 (± 5.6) |  | |
| *Only proportions are shown here because three of the four categories failed to achieve 50% posting rates, precluding calculation of median times. | | | | |
